# Overcoming language barriers with foreign-language speaking patients: a survey to investigate intra-hospital variation in attitudes and practices

**DOI:** 10.1186/1472-6963-9-187

**Published:** 2009-10-15

**Authors:** Patricia Hudelson, Sarah Vilpert

**Affiliations:** 1 Department of Community Medicine and Primary Care, Geneva University Hospitals, Rue Gabrielle-Perret-Gentil 4, 1211 Geneva 14, Switzerland

## Abstract

**Background:**

Use of available interpreter services by hospital clincial staff is often suboptimal, despite evidence that trained interpreters contribute to quality of care and patient safety. Examination of intra-hospital variations in attitudes and practices regarding interpreter use can contribute to identifying factors that facilitate good practice.

The purpose of this study was to describe attitudes, practices and preferences regarding communication with limited French proficiency (LFP) patients, examine how these vary across professions and departments within the hospital, and identify factors associated with good practices.

**Methods:**

A self-administered questionnaire was mailed to random samples of 700 doctors, 700 nurses and 93 social workers at the Geneva University Hospitals, Switzerland.

**Results:**

Seventy percent of respondents encounter LFP patients at least once a month, but this varied by department.

66% of respondents said they preferred working with ad hoc interpreters (patient's family and bilingual staff), mainly because these were easier to access. During the 6 months preceding the study, ad hoc interpreters were used at least once by 71% of respondents, and professional interpreters were used at least once by 51%.

Overall, only nine percent of respondents had received any training in how and why to work with a trained interpreter. Only 23.2% of respondents said the clinical service in which they currently worked encouraged them to use professional interpreters. Respondents working in services where use of professional interpreters was encouraged were more likely to be of the opinion that the hospital should systematically provide a professional interpreter to LFP patients (40.3%) as compared with those working in a department that discouraged use of professional interpreters (15.5%) and they used professional interpreters more often during the previous 6 months.

**Conclusion:**

Attitudes and practices regarding communication with LFP patients vary across professions and hospital departments. In order to foster an institution-wide culture conducive to ensuring adequate communication with LFP patients will require both the development of a hospital-wide policy and service-level activities aimed at reinforcing this policy and putting it into practice.

## Background

High quality, patient-centered care depends on doctors' listening to and understanding their patients' needs, and patients' understanding and following their doctors' advice [1]. In multicultural, multilingual contexts, language barriers present an important challenge to effective patient-provider communication [2-4].

Numerous studies have shown that quality of health care is compromised when foreign-language speaking patients who need linguistic assistance do not get interpreters [5-7]. It is also now well-established that more interpreter errors occur when untrained, ad hoc interpreters are used [8,9] and that trained professional interpreters positively affect foreign-language speaking patients' satisfaction, quality of care, and outcomes [10,11]. In some countries, such as the U.S. and Australia, the right to language access for foreign language speaking patients has been established through a range of national and state level legislation [12-14].

However, despite the scientific evidence and even in contexts with a favorable policy environment, use of trained interpreters is often suboptimal [15-18]. Even where trained medical interpreters are made widely available, healthcare providers may be unaware of existing services and their responsibility to use them, may not consider language access a priority issue, or there may be no system in place to ensure that health providers are held accountable for communication with foreign language speaking patients.

Such findings suggest that organizational culture may be important for understanding and changing clinical practices such as interpreter use [18-23]. For example, Waring et al. [24] found that hospital specialist departments' cultures shaped incident reporting practices, despite the existence of hospital-wide policy and guidelines. Their results suggest that identifying and understanding intra-institutional variation in attitudes and practices may be a prerequisite to improving institution-wide clinical practices. With regards to interpreter use, top-down policies and guidelines on when and why to use interpreters are necessary but probably insufficient to change clinical practices. Identifying conditions of "positive deviance" (in this case, staff that have adopted attitudes and practices conducive to good communication with LFP patients) may help to improve practices institution-wide [25,26].

To date most studies have been conducted in the USA and Australia [19]. Little is known about interpreter-use in European countries, and only a few studies have examined interpreter use within hospital systems [20,21]. While these studies suggest that the challenges are similar to those encountered in the USA, the European context differs in a number of significant ways. In most countries there is no national-level mandate requiring use of trained interpreters to communicate with foreign-language speaking patients and therefore institutional policies concerning language assistance vary considerably. In addition, many countries do not have access to professional telephone interpreting services, and the community interpreter profession is much less developed than in the USA. More research is needed to understand how the context of language barriers in health care affects attitudes and practices of health care professionals.

The purpose of our current study was to gain a representative picture of current attitudes, practices and preferences regarding communication with non-francophone patients at the Geneva University Hospitals, Switzerland, examine how these vary across professions and departments within the hospital, and identify factors associated with good practice. Results will be used to identify priority activities aimed at building an organizational culture that reflects concern for effective communication with foreign-language speaking patients.

## Methods

### Setting

Geneva University Hospitals (HUG) is a 2000-bed, public hospital group, organized into 11 medical departments, each containing 2 or more clinical services. The 11 departments include: Anesthesiology/Pharmacology/Intensive Care; Surgery; Child and Adolescent Health; Gynecology and Obstetrics; Community Medicine and Primary Care; Genetic Medicine and Laboratory; Internal Medicine; Clinical Neurosciences; Psychiatry; Rehabilitation and Geriatrics; and Imagery and Information Sciences. A full list of clinical services by department can be found on the HUG website [27].

The HUG provides care to a diverse population. In 2006, about 50% of patients were of non-Swiss nationality, representing 185 countries. To facilitate communication with foreign-language speaking patients, a community interpreter bank run by the Geneva Red Cross (GRC) has been available to all hospital personnel since 1999. Candidates (who generally have no prior interpreter training) are screened, hired and provided with an introduction to community interpreting by the GRC. Further training specific to medical interpreting is offered by the hospital, in the form of 2-hour seminars. A list of interpreters and their contact details is provided to the hospital, and is accessible to all staff via a hospital intranet site. The website provides guidelines on when and how to use an interpreter and offers training seminars for health care staff on request [28]. Staff members call the agency interpreters directly to make appointments, and interpreting is paid for by hospital departmental budgets. No professional telephone interpreting service is currently available to the HUG.

While no explicit hospital policy exists that mandates use of professional interpreters, in 2002 the hospital Clinical Ethics Committee took the position that " Even in the presence of a family member or friend who is well-disposed towards the patient, even if no conflict of interest exists between the patient and the institution that would put a [bilingual] health worker in an awkward position...one should systematically plan on using, at least initially, a mandated, professional interpreter." [29] Availability of professional interpreters is mentioned in the hospital brochure and in the information booklet given to hospitalized patients.

### Data collection methods

We developed a self-administered questionnaire consisting of 36 questions on respondents' sociodemographic and professional characteristics, frequency of contact with non-francophone patients, strategies and preferences regarding communication with these patients, training received and clinical service-level policies related to interpreter use, and opinions concerning priority activities for improving communication with non-francophone patients. The questionnaire was pretested with a convenience sample of 10 clinical colleagues to ensure the relevance and comprehensibility of the questions. The questionnaire was sent to the home address of study participants, and took approximately 10-15 minutes to complete. A second questionnaire was sent one month after the first mailing to all non-responders.

### Sampling

Sample size was determined in order to have sufficient statistical power (90%) and a low probability of type 1 error (5%), and to be able to detect between-group differences of 0.25 standard deviations (the exact number needed was 340). Given the habitually low-level participation of health professionals in mailed surveys, we expected a response rate of no more than 50%. Therefore, our initial sample size was 700 for doctors, 700 for nurses, and 93 for social workers (the total number working at the HUG). We excluded the Department of Imagery and Information Sciences from our sample due to their limited contact with patients.

### Analysis

Analysis focused on comparing respondents' attitudes, preferences and practices across hospital specialist departments, and exploring their association with factors such as frequency of contact with LFP patients, departmental instructions to staff about interpreter use, and training in why and how to work with an interpreter.

The study was funded by the Geneva University Hospitals quality programme. As a quality assessment project that entails minimal risk to participants, this study was exempted from review by the hospital research ethics committee.

## Results

### Respondent characteristics

Global response rate was 61% (doctors = 56%; nurses = 64%; social workers = 74%). All 10 hospital departments were represented in the final sample, but response rates varied by department, ranging from 50% (Dept of Genetic Medicine and Laboratory) to 69% (Dept of Anesthesiology, Pharmacology and Intensive Care).

### Frequency of contact with LFP patients

Seventy percent of respondents encounter LFP patients at least once a month (Table 1), but this varied by department. A majority of respondents from the Dept of Rehabilitation and Geriatrics never or rarely encountered LFP patients, while more than the half of respondents from the Dept of Community Medicine and Primary Care saw LFP patients more than eleven times a month. Overall, only 2.3% of respondents said they never encounter limited French-speaking (LFP) patients. The five patient-languages most frequently encountered during the last 6 months included English, Albanian, Portuguese, Spanish and Arabic.

**Table 1:** Frequency of contact with LFP patients

	**N**	**Percent**
Never	21	2.3
1-11 times per year	250	27.7
1-5 times per month	295	32.7
6-10 times per month	162	18.0
11-20 times per month	75	8.3
> 20 times per month	99	11.0
Total	902	100

### Strategies for overcoming language barriers

We asked respondents to indicate their preferred strategies for communicating with LFP patients, and to explain the reasons for their preferences. Overall, 66% preferred ad hoc interpreters (patient's family/friends, bilingual staff, children), while only 34% preferred professional interpreters (Table 2; total N is greater than 908 because some respondents chose more than one option). Preferences varied across departments: respondents from Psychiatry and Communitiy Medicine preferred GRC interpreters, while those from Clinical Neurosciences, Anesthesiology, Pharmacology and Intensive Care preferred bilingual staff (Table 3).

**Table 2:** Preferred strategies for communicating with LFP patients *

	**N**	**Percent**
Red Cross interpreters	324	34.2
Patient's family/friends	232	24.5
Children under 18 years of age	3	0.3
Bilingual hospital staff	387	40.9
Total	946	100.0

*Total N is greater than 908 because some respondents chose more than one option

**Table 3:** Language strategy preferences by department

**Language strategy preferred**	**Anesthesiology/** **Pharmacology/** **Intensive Care**	**Surgery**	**Child and Adolescent Health**	**Gynecology and Obstetrics**	**Community Medicine and Primary Care**	**Genetic Medicine and Laboratory**	**Internal Medicine**	**Clinical Neurosciences**	**Psychiatry**	**Rehabilitation and Geriatrics**	**χ**^**2**^	**p <**	**V**
GRC interpreters	15 (16.67%)	16 (17%)	68 (50%)	14 (45%)	43 (60%)	4 (20%)	38 (28%)	10 (17%)	93 (56%)	23 (23%)	112.621	0.000	0.352
Patient's family/friends	28 (31%)	37 (39%)	18 (13%)	6 (19%)	13 (18%)	6 (30%)	43 (32%)	25 (42%)	21 (13%)	34 (33%)	53.518	0.000	0.243
Bilingual hospital staff	48 (53%)	44 (46%)	54 (39%)	11 (35%)	18 (25%)	10 (50%)	59 (43%)	28 (47%)	62 (37%)	52 (51%)	20.862	0.013	0.152

**Figure 1:**
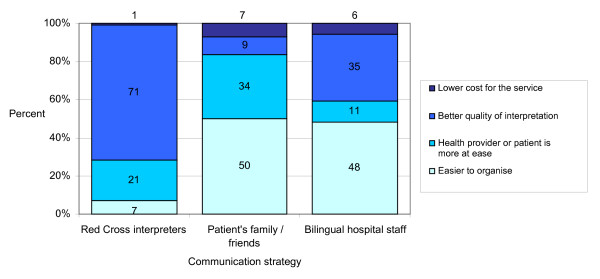
**Reasons for preference of communication strategy**.

Reasons for respondents' preferences differed according to which method was favored (Figure 1). Those who preferred GRC interpreters appreciated their professionalism (translation quality and confidentiality), whereas those who preferred ad hoc interpreters highlighted their practical advantages (immediate availability, easier to organize).

**Figure 2:**
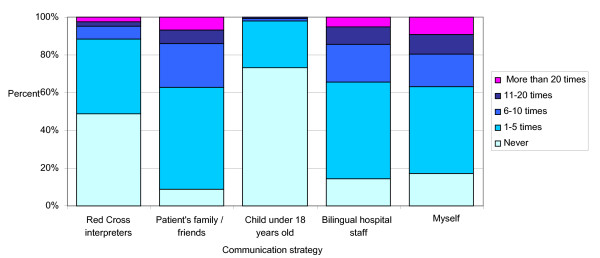
**Strategies used to overcome language barriers and frequency of use in the last 6 months**.

We also asked respondents to indicate the strategies they actually used over the last six months. Ad hoc interpreters (patient's family/friends, hospital personnel, children) were used the most often (Figure 2), at least once by 71% of respondents, while GRC interpreters were used at least once by 51%. The Departments of Community Medicine and Primary Care and Gynecology and Obstetrics had the highest level of GRC interpreter use: 31% and 27% of respondents, respectively, had used the service at least once during the previous 6 months. (Table 4)

**Table 4:** Use of GRC interpreters during previous 6 months, by department

**Use of GRC interpreters during previous 6 months**	**Anesthesiology/** **Pharmacology/** **Intensive Care**	**Surgery**	**Child and Adolescent Health**	**Gynecology and Obstetrics**	**Community Medicine and Primary Care**	**Genetic Medicine and Laboratory**	**Internal Medicine**	**Clinical Neurosciences**	**Psychiatry**	**Rehabilitation and Geriatrics**
Never	57 (74%)	61 (69%)	34 (26%)	4 (15%)	22 (33%)	10 (59%)	63 (50%)	28 (53%)	48 (33%)	70 (85%)

1-5 times	19 (25%)	26 (29%)	74 (57%)	15 (58%)	24 (36%)	6 (35%)	53 (42%)	22 (42%)	72 (50%)	10 (12%)

6 and more	1 (1%)	2 (2%)	22 (17%)	7 (27%)	21 (31%)	1 (6%)	9 (7%)	3 (5%)	25 (17%)	2 (2%)

χ^2 ^= 168.523; p < 0,000; dl 18

λ = 0.186, dependent variable "Use of GRC interpreters during previous 6 months"

V = 0,322; P < 0,000

The common recourse to bilingual staff is also reflected in the results of a separate item on the questionnaire which asked if respondents had ever themselves interpreted for a patient. 52.2% of respondents reported having interpreted for a patient at some time in the past, most often for English, German, Spanish and Italian.

Finally, a large proportion of respondents (83%) communicated directly with their patients in a language other than French more than once during the last six months (Figure 2), although we do not have data on whether they communicated in the patient's primary language or in a third language.

### Training

Respondents are generally poorly prepared to ensure adequate communication with LFP patients. Only 9% of respondents had received any training in how and why to work with a trained interpreter. Nurses are the least prepared, with only 4.5% reporting having received any training (Table 5). The percent of staff having received training varied across departments (Table 6), with the highest levels found in the departments of Community Medicine (37.5%) and Psychiatry (14.5%). These are the only 2 departments that have incorporated training on how and why to work with an interpreter into their postgraduate training programs for residents.

**Table 5:** Percent of respondents having received training in how to work with an interpreter

**Training**	**Doctor**	**Nurse**	**Social worker**
Yes	56 (14.3%)	20 (4.5%)	9 (13.2%)
No	336 (85.7%)	423 (95.5%)	59 (86.8%)
Total N	392	443	68

χ^2 ^= 24.546; p < 0,000; dl 2

V = 0,165; P < 0,000

**Table 6:** Training in how/why to use an interpreter by department

**Received training**	**Anesthesiology/** **Pharmacology/** **Intensive Care**	**Surgery**	**Child and Adolescent Health**	**Gynecology and Obstetrics**	**Community Medicine and Primary Care**	**Genetic Medicine and Laboratory**	**Internal Medicine**	**Clinical Neurosciences**	**Psychiatry**	**Rehabilitation and Geriatrics**
Yes	2 (2%)	3 (3%)	13 (10%)	1 (3%)	27 (38%)	0	9 (7%)	3 (5%)	24 (15%)	3 (3%)

No	88 (98%)	92 (97%)	122 (90%)	30 (97%)	45 (63%)	20 (100%)	126 (93%)	56 (95%)	141 (85%)	97 (97%)

TOTAL N	90	95	135	31	72	20	135	59	165	100

### Departmental policies and their influence on respondent attitudes

Instructions to staff about communicating with LFP patients were not uniform throughout the hospital (Table 7). Overall, only 23.2% of respondents said the clinical service in which they currently worked encouraged them to use the GRC interpreter service to facilitate communication with LFP patients. 12.1% said they were told to use ad hoc interpreters and to call the GRC interpreter service only as a last resort. 64.7% said they were given no information at all about communicating with LFP patients. Encouragement to use GRC interpreters was reported most frequently by respondents from the Department of Community Medicine (56%).

**Table 7:** Respondents' reports of messages to staff about interpreter use, by department

**Message to staff**	**Anesthesiology/Pharmacology/** **Intensive Care**	**Surgery**	**Child and Adolescent Health**	**Gynecology and Obstetrics**	**Community Medicine and Primary Care**	**Genetic Medicine and Laboratory**	**Internal Medicine**	**Clinical Neurosciences**	**Psychiatry**	**Rehabilitation and Geriatrics**
Encourages use of GRC interpreters	4 (5%)	4 (4%)	59 (44%)	5 (16%)	38 (56%)	1 (5%)	19 (14%)	6 (11%)	63 (39%)	7 (7%)
										
Encourages use of alternative strategies	9 (10%)	14 (15)	12 (9%)	5 (16%)	5 (7%)	0	12 (9%)	3 (5%)	33 (21%)	13 (13%)
										
Provides no guidance	74 (85%)	76 (81)	64 (47%)	21(68%)	25 (37%)	19 (95%)	105 (77%)	48 (84%)	64 (40%)	78 (78%)
										
Total N	87	94	135	31	68	20	136	57	160	98

Respondents working in services where use of GRC interpreters was encouraged were more likely to be of the opinion that the hospital should systematically provide a professional interpreter to LFP patients (40.3%) as compared with those working in a department that discouraged use of GRC interpreters (15.5%) (Table 8); they also used GRC interpreters more often during the previous 6 months (Table 9). Indeed, respondents working in services where use of GRC interpreters is encouraged were on average two times more likely to have used a GRC interpreter during the past 6 months than respondents working in a service where there is no encouragement or instruction.

**Table 8:** Respondents' opinions about use of GRC interpreters by service-level policy concerning communication with LFP patients

	**Service**		
	
**Opinion about use of GRC interpreters**	**Encourages GRC use**	**Discourages GRC use**	**Provides no guidance**
Systematically	40.3%	15.5%	16.2%
In some situation	45.3	39.8	47.1
Only when no other solution	14.4	44.7	36.7
Total %	100	100	100
(N)	201	103	556

χ^2 ^= 70,517; p < 0,000; dl 4

V = 0,202; P < 0,000

**Table 9:** GRC interpreter use by service-level policy about communication with LFP patients

	**Service**		
	
**Use of GRC interpreters during last 6 months**	**Encourages GRC use**	**Discourages GRC use**	**Provides no guidance**
Never	13.2%	57.5%	60.7%
1-10 times	72.6	42.5	37.2
11+ times	14.2	0.0	2.1
Total %	100	100	100
(N)	197	87	514

χ^2 ^= 153,696; p < 0,000; dl 4

λ = 0.285, dependent variable "Use of GRC interpreters during previous 6 months"

V = 0,310; P < 0,000

## Discussion

We found that doctors, nurses and social workers at our hospital had frequent contact with LFP patients. However, they did not generally consider recourse to professional interpreters to be a priority and respondents were unprepared to ensure adequate communication with LFP patients. Not surprisingly, strategies for overcoming language barriers are suboptimal. Most respondents preferred using ad hoc interpreters, and use of bilingual staff was particularly common.

Nonetheless, positive attitudes and practices were identified in some departments and services, indicating that conditions can be created that foster adequate communication with LFP patients. Over a third of respondents preferred working with professional interpreters and recognized their benefits in terms of confidentiality and quality of interpreting. Furthermore, a fifth of respondents thought that the hospital should systematically use professional interpreters to communicate with non-francophone patients, and one-half of respondents had used a GRC interpreter at least once during the previous 6 months. Respondents that had received training or worked in departments that actively encouraged use of professional interpreters were more likely to think that the hospital should systematically use professional interpreters, were more likely to have used a professional interpreter and to prefer them. This suggests that creating a positive practice environment is important for influencing behavior change, and that it may be possible to encourage good practices from the botton-up even in contexts where there is no top-down, hospital-wide mandate to use professional interpreters.

Nonetheless, the challenges to ensuring adequate language assistance for foreign-language speaking patients remain daunting. A number of studies suggest that despite hospital, state and national legislature aimed at ensuring access to professional medical interpreters, use remains inadequate [30,31]. Burbano et al[32] found that even though pediatric residents unanimously agreed that hospital interpreters were effective, actual use was low. Residents tended to rely on their own inadequate language skills or on bilingual staff to interpret for them. Similarly, Diamond et al. [18] found that, despite misgivings about the implications for quality of care, residents preferred to "get by" with ad hoc interpreters or none at all. Time pressures and limited interpreter availability are frequently cited as reasons for underuse of professional interpreters, but underestimates of patient and physician language proficiency may also play a role. In our context, cost control pressures may be an additional disincentive to using professional interpreters. The hospital is currently undergoing a multi-year budget cutting exercise which has put pressure on departments to control costs. The effects on attitudes and practices related to interpreter use are unknown, but financial pressures are likely to act as an important deterrent to use of GRC interpreters. A study carried out in 2004 involving a small, convenience sample of doctors and nurses at the HUG [33] suggested that professional interpreters were called in only when ad hoc interpreters (family or hospital staff) were unavailable. Data are lacking to explain these findings, but cost concerns and scheduling difficulties were mentioned by some respondents as reasons for not calling a GRC interpreter. In our current study, cost concerns were mentioned only infrequently by respondents as a reason for preferring ad hoc interpreters, and only 12% of respondents said their departments actively discouraged GRC interpreter use, but cost-containment pressures may have a more subtle influence in clinical practice, and in fact anecdotal evidence suggests a generalized reluctance to incur additional costs to departments by using professional interpreters.

We have no data on the adequacy of language assistance in different situations, but it may be that not all situations where ad hoc interpreters are used are characterized by inadequate language assistance. More in depth research is needed to explore these issues [34,35], and as Hsieh has argued [36] the ultimate challenge is for clinicians to be able to distinguish between situations where a professional interpreter is essential and those where ad hoc interpreters may be sufficient, and act accordingly.

In our study we found that few departments provide information to staff on when and how to work with professional interpreters, which reinforces the sentiment that their use is optional and not essential for quality care. Changes in clinical practice are unlikely in an environment where the unspoken message is that ad hoc interpreters are "good enough" for most situations, and that professional interpreters are only needed when other methods are unavailable.

Our study does not provide data on exactly which service-level practices lead to positive attitudes and practices, but we agree with Diamond et al. [18] who believe that increasing professional interpreter use will require not only interventions at the level of individual clinicians (training, guidelines) but also at the level of the practice environment, including norms, structural changes, and role models. In the Department of Community Medicine and Primary care where interpreter use is the norm, an articulated, shared mission to provide quality care to diverse patients, positive role models from senior staff, and systematic training of new interns in when and how to work with interpreters all contribute to creating a "service culture" conducive to ensuring adequate communication with LFP patients.

The challenge is to spread this positive "service culture" to the rest of the hospital. While activities aimed at facilitating access to professional interpreters will be important (systematic patient-language data collection; a central number for requesting an interpreter; telephone interpreting, etc.), these alone cannot create an institutional culture favorable to interpreter use. Based on our results, we believe that other priority activities will include developing an explicit hospital policy statement on interpreter use (when, why and how interpreters should be called), and communicating this policy during orientation of all new staff. Specific, service-level activities will also be needed to reinforce this policy, and put it into practice. Senior role models, systematic training of staff and visible information in clinical services about interpreter services (rights of patients, contact information, etc.) will also be important for influencing institutional culture. Finally, evaluation and feedback to clinical services about their performance with regards to communicating with and caring for LFP patients will help to change perceptions of interpreter use from an optional activity to that of a quality indicator.

Our study contributes to the scarce literature on language barriers in health care in Europe. However, it was conducted in a single Swiss hospital, and therefore our conclusions may not be generalizable to other settings. Furthermore, small numbers prevented more detailed analyses of factors affecting respondents' attitudes and practices. Finally, questionnaire data can only suggest general attitudes and motivations of respondents. A more in-depth, qualitative look at service-level attitudes and practices would contribute to a better understanding of the factors and conditions associated with good practice.

## Conclusion

Attitudes and practices regarding communication with LFP patients vary across professions and hospital departments. In order to foster an institution-wide culture conducive to ensuring adequate communication with foreign-language speaking patients, both hospital-wide policy and service-level activities aimed at reinforcing this policy and putting it into practice will be necessary.

## Competing interests

The authors declare that they have no competing interests.

## Authors' contributions

PH conceived the study, designed the questionnaire, and drafted the manuscript; SV contributed to the design of the questionnaire, carried out the data collection and analysis, and contributed to the manuscript. Both authors read and approved the final manuscript.

## Authors' information

Patricia Hudelson, PhD, is a medical anthropologist. Based in the Department of Community Medicine and Primary Care, she teaches and conducts research in the area of cross cultural care and communication. Since 2006 she also oversees interpreter use within the hospital, and in this role develops research and training activities aimed at ensuring effective communication with foreign-language speaking patients.

Sarah Vilpert, MA, has training in both sociology and demography. As a research assistant within the Department of Community Medicine and Primary Care, she has been involved in assessing language assistance needs in the hospital as well as in the evaluation of a cultural consultation service.

## Pre-publication history

The pre-publication history for this paper can be accessed here:


